# Income Tax of Physicians of the 30th Dist. of New York for 1862

**Published:** 1864-03

**Authors:** 


### INCOME TAX OF PHYSICIANS OF THE 30TH DIST. OF NEW YORK FOR 1862.

With the view of showing the amount contributed to Internal Revenue by the Physicians of the 30th District, we called upon Dr. O. F. Presbrey, U. S. Assessor, who kindly consented to give us the following very general facts; amounts returned by individuals are not divulged unless there are suspicions of fraud:

Ninety-six physicians in Buffalo took license; 75 physicians in the other towns in Erie County; 54 physicians in the City division made return of an income derived exclusively from professional services of over $600 per annnm. Of these, twelve were in receipt of over $600 and under $1000; twenty-two received over $1000 and under $2000; seven received over $2000 and under $3000; eight were in receipt of over $3000 and under $4000; four of over $4000 and under $5000; one received over $5000—$5500. These figures apply to the city physicians, many of whom return incomes in addition from other sources—rents, bonds and mortgages and other securities.

This shows the amounts received. From it is deducted $600 and the expenses incident to doing the business, etc., and a tax of 3 per cent is paid upon the remainder.

Thirty physicians receive sufficient income to pay income tax, the smallest of which is 71 cents and the largest tax including income from all sources is $205.49.

These figures are instructive and very suggestive. It proves that medicine is not practiced for money; that it is not productive of anything like a great income even under the most favorable circumstances; while in the great majority of cases it barely affords a subsistence. Out of 171 physicians not more than 35 or 40 receive more than the law allows as necessary for subsistence and exempt from taxation. Of these only 20 receive over $2000, and not a single one in Erie county can be said to receive a large professional income. After publishing these statistics from the Assessor, we predict that Buffalo will not be regarded as a desirable field for physicians to cultivate; it has, however. some redeeming features after all. There is not great differences in the receipts of physicians; it is told in few words. One-quarter in the district receive over $600 and the others less; consequently there is a general level and no summits upon which to bulid aristocratic pretensions. Again it is a field where physicians are not obliged to contribute largely to Internal Revenue, and may be looked upon favorably on that account. In view of the above facts, it would seem that the time of physicians in any single instance cannot be wholly absorbed in professional duty, and that the cultivation and advancement of the science of medicine might receive some attention from all. Hereafter, when we look for, and invite contributions for the Journal, and are told, "it is impossssible to get a minute's time for such purpose, otherwise it should most certainly be done, and with the greatest pleasure," we shall only have to turn to our statistics to see that either Internal Revenue, or ourselves, are greatly defrauded. Physicians who only receive 5000 or 6000, and this mostly for services at the highest prices, can do much more, if it is offered; they must have time which if devoted to that purpose, would enable them to contribute something to the sum of medical knowledge.

We had supposed that $10,000 or $12,000 income would be offered as apology, and had come to consider *it valid*, knowing that money was no stimulus to exertion unless it induce greater efforts to obtain more; but when we see $5500 is the maximum income, we conclude that "*want of time*" is not the reason it is so difficult for men of experience and culture, to make contributions to medical literature. It is generally true that men who do the most professional labor, also have most time for literary pursuits, and as frequent that those who plead "no time to spare" actually spare the most time.

